# Genome Sequence of *Paenibacillus* sp. Strain FJAT-28004 for the Genome Sequencing Project for Genomic Taxonomy and Phylogenomics of *Bacillus*-Like Bacteria

**DOI:** 10.1128/genomeA.00863-15

**Published:** 2015-10-22

**Authors:** Guo-hong Liu, Bo Liu, Jie-ping Wang, Jian-Mei Che, Yu-Jing Zhu, Qian-Qian Chen, Chuan-Qing Ruan

**Affiliations:** Agricultural Bio-Resources Research Institute, Fujian Academy of Agricultural Sciences, Fuzhou, Fujian, China

## Abstract

*Paenibacillus* sp. strain FJAT-28004 is a spore forming and strictly aerobic bacterium. Here, we report the draft 7,479,858-bp genome sequence of *Paenibacillus* sp. FJAT-28004, which will provide useful information for genomic taxonomy and phylogenomics of the genus *Paenibacillus*, as well as for the functional gene mining and application of *Paenibacillus* sp. FJAT-28004.

## GENOME ANNOUNCEMENT

The genus *Paenibacillus* was created from the group 3 bacilli on the basis of a 16S rRNA gene sequence analysis conducted by Ash et al. (1). Since the description of the genus, species belonging to the genus *Paenibacillus* have been isolated from various ecological habitats, including warm springs (2), rice fields (3), alkaline soils (4), and the phyllospheres and rhizospheres of trees (5). During the survey of the diversity of bacteria in Xizang, *Paenibacillus* sp. strain FJAT-28004 was isolated from the dry deadwood sample collected from Nyingchi prefecture in Xizang, China. From the 16S rRNA phylogenetic analysis, it was observed that strain FJAT-28004 was clearly distinguishable from other *Paenibacillus* species and should be a new species of the genus *Paenibacillus*. In order to confirm this, FJAT-28004 was selected as one of the research objects in our “genome sequencing project for genomic taxonomy and phylogenomics of Bacillus-like bacteria.”

The genome sequencing of FJAT-28004 was performed via the Illumina Hiseq2500 system. Three different DNA libraries with insert sizes of 180, 500,  and 3K bp were constructed and sequenced using the 2- × 125-bp paired-end sequencing strategy. A total of 4,417,476,776 bp of clean sequence data were obtained, consisting of 17,669,907 reads, providing approximately 735-fold coverage. The reads were assembled via the SOAPdenovo software version 1.05 (6), using the key parameter K setting at 17. Through the data assembly, 21 scaffolds were obtained, and the scaffold *N*_50_ was 5,163,634 bp. The average length of the scaffolds was 356,183 bp, and the longest and shortest scaffolds were 5,163,634 bp and 1,086 bp, respectively.

The annotation of the genome was performed using the NCBI Prokaryotic Genomes Automatic Annotation Pipeline (PGAAP) utilizing GeneMark, Glimmer, and tRNAscan-SE tools (7). A total of 7,033 genes were predicted, including 6,322 functional genes, 119 pseudo genes, 5 miRNAs, 72 tRNAs, and 6 rRNA genes. Also, 1 clustered regularly interspaced short palindromic repeat (CRISPR) array was found in the draft genome. The average DNA G+C content was 44.3%.

From the genome sequence analysis, FJAT-28004 was predicted to possess complete carbon metabolic pathways, including those for glycolysis, the citrate cycle (tricarboxylic acid [TCA] cycle), and the pentose phosphate pathway. In particular, *Paenibacillus* sp. FJAT-28004 was predicted to be equipped with a wide variety of genes for amino acid and organic acid metabolism, agreeing with the physiological properties of FJAT-28004.

### Nucleotide sequence accession numbers.

This whole-genome shotgun project has been deposited at DDBJ/EMBL/GenBank under the accession no. LGHP00000000. The version described in this paper is version LGHP00000000.1.
